# Engineering *Escherichia coli* for increased Und-P availability leads to material improvements in glycan expression technology

**DOI:** 10.1186/s12934-024-02339-8

**Published:** 2024-03-01

**Authors:** Emily J. Kay, Manoj K. Dooda, Joseph C. Bryant, Amanda J. Reid, Brendan W. Wren, Jerry M. Troutman, Matthew A. Jorgenson

**Affiliations:** 1 Department of Infection Biology, London School of Hygiene and Tropical Medicine, London, WC1E 7HT UK; 2 Department of Biological Sciences, University of North Carolina at Charlotte, Charlotte, NC 28223 USA; 3 Department of Microbiology and Immunology, University of Arkansas for Medical Sciences, 4301 West Markham St. / Biomed I, Room 511 / Little Rock, Little Rock, AR 72205 USA; 4 Nanoscale Science Program, University of North Carolina at Charlotte, Charlotte, NC 28223 USA; 5 Department of Chemistry, University of North Carolina at Charlotte, Charlotte, NC 28223 USA

**Keywords:** Undecaprenyl phosphate, Polysaccharide, Capsule, Glycoengineering

## Abstract

**Background:**

Bacterial surface glycans are assembled by glycosyltransferases (GTs) that transfer sugar monomers to long-chained lipid carriers. Most bacteria employ the 55-carbon chain undecaprenyl phosphate (Und-P) to scaffold glycan assembly. The amount of Und-P available for glycan synthesis is thought to be limited by the rate of Und-P synthesis and by competition for Und-P between phosphoglycosyl transferases (PGTs) and GTs that prime glycan assembly (which we collectively refer to as PGT/GTs). While decreasing Und-P availability disrupts glycan synthesis and promotes cell death, less is known about the effects of increased Und-P availability.

**Results:**

To determine if cells can maintain higher Und-P levels, we first reduced intracellular competition for Und-P by deleting all known non-essential PGT/GTs in the Gram-negative bacterium *Escherichia coli* (hereafter called ΔPGT/GT cells). We then increased the rate of Und-P synthesis in ΔPGT/GT cells by overexpressing the Und-P(P) synthase *uppS* from a plasmid (p*uppS*). Und-P quantitation revealed that ΔPGT/GT/p*uppS* cells can be induced to maintain 3-fold more Und-P than wild type cells. Next, we determined how increasing Und-P availability affects glycan expression. Interestingly, increasing Und-P availability increased endogenous and recombinant glycan expression. In particular, ΔPGT/GT/p*uppS* cells could be induced to express 7-fold more capsule from *Streptococcus pneumoniae* serotype 4 than traditional *E*. *coli* cells used to express recombinant glycans.

**Conclusions:**

We demonstrate that the biotechnology standard bacterium *E*. *coli* can be engineered to maintain higher levels of Und-P. The results also strongly suggest that Und-P pathways can be engineered to increase the expression of potentially any Und-P-dependent polymer. Given that many bacterial glycans are central to the production of vaccines, diagnostics, and therapeutics, increasing Und-P availability should be a foremost consideration when designing bacterial glycan expression systems.

**Supplementary Information:**

The online version contains supplementary material available at 10.1186/s12934-024-02339-8.

## Background

Enveloped bacteria express a wide array of polysaccharides at the cell surface that confer morphology, protect against environmental insults, and resist killing by immune systems [1]. Most of these polysaccharides are linked to proteins or lipids and are referred to as glycoconjugates, a diverse class of molecules that include glycoproteins, lipopolysaccharides, capsular polysaccharides, lipoarabinomannans, peptidoglycan (PG), glycosylated teichoic acids, and many other clinically important surface structures [2]. The precursors that form these layers are assembled on an essential lipid carrier known as undecaprenyl phosphate (Und-P), which is also referred to as bactoprenyl phosphate [3]. Und-P is a 55-carbon isoprenoid that is first synthesized as a diphosphate (Und-PP) on the inner face of the cytoplasmic membrane by the undecaprenyl pyrophosphate synthase (UppS) via the methylerythritol phosphate (MEP) pathway [4, 5]. Und-PP is also generated on the outer face of the cytoplasmic membrane when it is released during glycan polymerization. Und-PP is then dephosphorylated by Und-PP pyrophosphatases to Und-P. The integral membrane pyrophosphatase BacA and PAP2 family proteins dephosphorylate Und-PP [6–8] and several lines of evidence indicate that this activity occurs on the outer face of the cytoplasmic membrane [8–11]. Since Und-PP is synthesized on the inner face of the cytoplasmic membrane, enzymes possessing Und-PP pyrophosphatase activity would appear to be required on both sides of the cytoplasmic membrane. However, a cytoplasmic Und-PP phosphatase has yet to be discovered.

Once Und-P is active, phosphoglycosyl transferases (PGTs) catalyze the transfer of phosphosugars from nucleoside diphosphate sugar donors to Und-P to form Und-PP-linked sugar monomers [12], which are progressively built into oligosaccharide building blocks by other GTs. GTs also catalyze the transfer of sugars to Und-P to form Und-P-linked sugar monomers that serve as donors to other glycans [13, 14]. Collectively, we refer to these priming enzymes as PGT/GTs. Since most bacteria express multiple Und-P-dependent glycans, cells usually encode several PGT/GTs. For example, the Gram-negative bacterium *Escherichia coli*, which is the workhorse of the biotechnology industry, harbors five PGT/GTs [Enzyme, product]: MraY, PG; WecA, O-antigen and enterobacterial common antigen (ECA); WcaJ, colanic acid capsule; ArnC, aminoarabinose modification of phosphate groups on Lipid A; GtrB, glucose modification of O-antigen. We note that most lab strains of *E*. *coli* do not produce O-antigen due to an insertion in *wbbL* (K-12 derivatives) or *wbbD* (B derivatives) [15, 16]. Of these PGT/GTs, only MraY is essential, so that mutants lacking individual PGT/GTs are readily obtained [17]. Recently, we discovered that deleting *wecA* increases the free pool of Und-P in *E*. *coli* [18]. This finding prompted us to determine if other Und-P pathways could be modified to increase the pool of Und-P.

The present results show that *E*. *coli* can be induced to maintain at least three times more Und-P than wild type cells by overexpressing the Und-PP synthase *uppS* in cells lacking all non-essential PGT/GTs. That Und-P levels can rise to such a degree is notable given that an overabundance of polyprenyl phosphates like Und-P is thought to destabilize phospholipid membranes [19]. Since increasing Und-P levels increases production of Und-PP-linked intermediates [18, 20, 21], we reasoned that similar effects might occur for the production of finished glycans. Indeed, increasing cellular Und-P levels increased both endogenous and heterologous glycan expression in *E*. *coli*. In particular, we found that recombinant *Streptococcus pneumoniae* capsular polysaccharide expression (important for the production of bioconjugate pneumococcal vaccines [22–24]) dramatically rises in cells containing more Und-P. Since Und-P is a universal carrier lipid, the results suggest similar increases may occur for potentially any Und-P-dependent polymer. Thus, increasing Und-P availability should be considered a prime driver for systems used to express bacterial glycans. Such processes (termed Glycan Expression Technology) are currently underpowered by low glycan yields [25–29].

## Results

### The free pool of Und-P increases in a Und-P pathway-minimized strain

Previously, we constructed a mutant lacking WecA in the *E*. *coli* strain MG1655 and found these cells to contain more Und-P [18]. Since deleting *wecA* prevents formation of ECA in this strain background, we sought to determine if similar effects would occur for other Und-P-using pathways. To this end, we systematically disrupted all non-essential Und-P-dependent pathways in *E*. *coli*. However, since MG1655 cells do not produce O-antigen due to insertion sequences in *wbbL* [15], we engineered pathway mutants in MG1655 *wbbL* + cells (hereafter referred to as wild type [WT] cells) [30]. Pathway mutations were introduced at the point of initiation to prevent the accumulation of Und-PP-linked intermediates, which lower Und-P levels [18]. Thus, we individually deleted *wecA* (ECA and O-antigen), *wcaJ* (colanic acid), *gtrB* (O-antigen glucosylation), and *arnC* (modification of lipid A with aminoarabinose). Since WecA initiates ECA and O-antigen synthesis in MG1655 derivatives, we specifically prevented ECA expression (but not O-antigen) by deleting the *wecB* epimerase, which is required to produce the second building block in ECA synthesis (i.e., UDP-*N*-acetylmannosaminuronate) [31]. Importantly, WecA^+^WecB^−^ cells are not expected to sequester Und-P in Und-PP-linked ECA intermediates (i.e., they will not lower Und-P levels). Similarly, the *wbbL*::IS*5* insertion mutation prevents the formation of the second building block in O-antigen synthesis (i.e., dTDP-L-rhamnose) without sequestering Und-P [32].

To ensure our mutations did not inadvertently lower Und-P levels, we first examined our pathway mutants for changes in morphology (i.e., PG synthesis), which is highly responsive to reductions in Und-P availability [32–34]. As expected, pathway mutants produced rod-shaped cells (Fig. S1A) that were similar in size to wild type cells when examined by flow cytometry (Fig. S1B). We note that cells lacking O-antigen (i.e., *wbbL*::IS*5* and Δ*wecA*) grew slightly larger, confirming its role as a mechanical element within the cell envelope [35]. In short, our cell morphology results indicated that Und-P levels were not reduced in our pathway mutants.

**Fig. 1:**
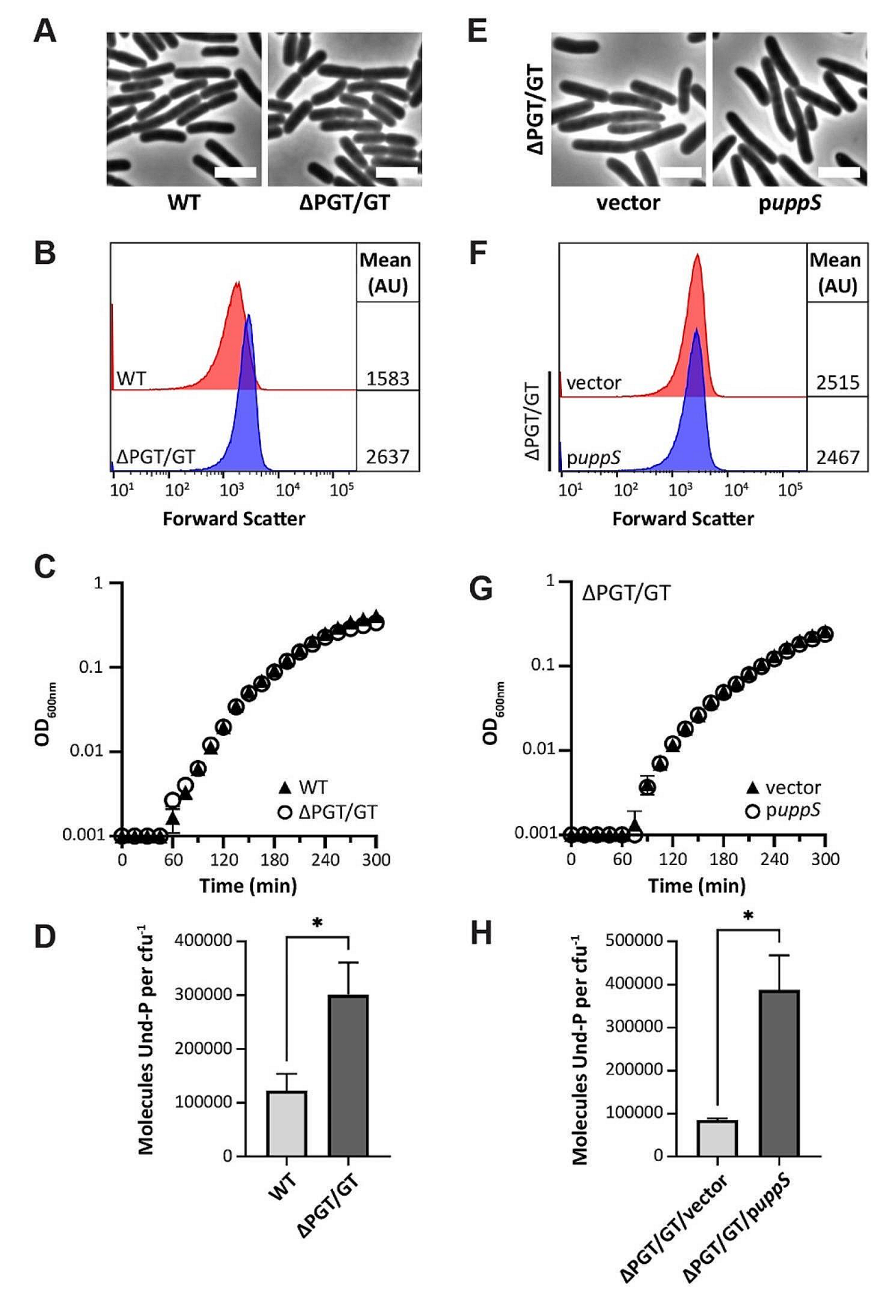
Maximizing Und-P levels in a Und-P pathway-minimized strain of *E*. *coli*. (**A** and **E**) Micrographs of cells with the indicated genotypes. Cells were grown in TB (panel A) and TB containing 500 µM IPTG (panel E) at 37 °C until the culture reached an OD_600_ of 0.4 to 0.6. The cells were then photographed by phase contrast microscopy. Bar, 3 μm. (**B** and **F**) Flow cytometry data from live cells in panels A and E. Histograms of the forward scatter area from 100,000 cells are shown. The mean cell size is shown in arbitrary units (AU). (**C** and **G**) Growth curves for cells with the indicated genotypes cultured at 37 °C in TB (panel C) or TB containing 500 µM IPTG (panel G). Error bars show +/- standard deviation of the means. (**D** and **H**) Und-P levels from cells grown in panel A (after 3.5 h) and panel E (after 24 h). Und-P levels were normalized by dividing Und-P measurements by the mean CFU/ml. Absolute Und-P values are detailed in Table S1. Additional Und-P pathway mutants are shown in Fig. S1. Error bars show +/- standard error of the means. Significance was determined by using an unpaired *t*-test followed by Welch’s correction. *p < 0.05. Morphological data are representative of two independent experiments. Growth and Und-P measurements are representative of two independent experiments performed in triplicate. The *E*. *coli* strains shown are MAJ330 (WT), MAJ557 (ΔPGT/GT), MAJ1385 (ΔPGT/GT/vector), and MAJ1386 (ΔPGT/GT/p*uppS*).

To determine if Und-P levels had increased in our pathway mutants, we measured Und-P levels directly by liquid chromatography – mass spectrometry (LC-MS). We note that wherever Und-P levels are discussed, they only include the free pool of Und-P and not those Und-P molecules linked to glycan intermediates. Intracellular quantitation of Und-P levels revealed that WT cells harbor on average 123,000 molecules of Und-P per cell (Fig. S1C and Table S1), which was consistent with previously measured values [36]. To our surprise, similar levels of Und-P were also obtained for mutants lacking individual pathways and WecA, which is ECA and O-antigen minus. Since multiple Und-P-utilizing pathways were still active in our pathway mutants, and since Und-P pathways compete for a common pool of Und-P [32, 33, 37], the observed results suggested that Und-P was being redirected in our strain backgrounds. Therefore, we engineered an Und-P pathway-minimized strain of *E*. *coli* lacking WecA, WcaJ, GtrB, and ArnC, which we refer to as ΔPGT/GT cells. Morphological analysis revealed that ΔPGT/GT cells produce rod-shaped cells that are somewhat larger than WT (Fig. 1A and B), consistent with the absence of O-antigen. Further analysis revealed that ΔPGT/GT cells grow identical to wild type cells in rich media (Fig. 1C). Interestingly, Und-P quantitation revealed that ΔPGT/GT cells harbor on average 301,000 molecules of Und-P per cell, which represents a 145% increase in the free pool of Und-P when compared to WT cells (Fig. 1D and Table S1, compare WT to ΔPGT/GT cells). These results indicate that ~ 175,000 molecules of Und-P are (collectively) employed by WecA, WcaJ, GtrB, and ArnC when cultured under standard laboratory conditions. In summary, our results demonstrate that *E*. *coli* can be engineered to express fewer Und-P-utilizing pathways. Our results also demonstrate that *E*. *coli* cells can tolerate increases in the free pool of Und-P.

### Increasing Und-P in a Und-P-pathway minimized strain

At this point, we sought to determine if ΔPGT/GT cells could be induced to maintain even higher levels of Und-P. When *E*. *coli* is cultured under aerobic growth, the Und-PP synthase UppS competes with IspB for the isoprenoid precursors IPP (isopentenyl pyrophosphate) and FPP (farnesyl pyrophosphate) [38, 39]. Since Und-PP synthesis is limited by substrate competition, we reasoned that overexpressing *uppS* would promote Und-P availability in ΔPGT/GT cells, presumably by limiting the flux of isoprenoid precursors to IspB. Therefore, we transformed ΔPGT/GT cells with a plasmid expressing an IPTG-inducible copy of *uppS* (p*uppS*). As can be seen in Fig. 1, *uppS* overexpression had no effect on the shape (Fig. 1E and F) or growth (Fig. 1G) of ΔPGT/GT cells. Subsequent Und-P quantitation revealed that ΔPGT/GT/p*uppS* cells could be induced to contain on average 387,000 molecules of Und-P per cell (Fig. 1H and Table S1), which represents a 29% and 215% increase in the free pool of Und-P when compared to ΔPGT/GT and WT cells, respectively. Interestingly, ΔPGT/GT/vector cells contained on average 100,000 molecules of Und-P per cell, far below the expected 300,000 molecules per cell we observed in ΔPGT/GT cells. Although we cannot fully explain this result, we are currently investigating the effect of plasmid suppression on Und-P levels. In any event, our results suggest that UppS activity bottlenecks Und-P availability and that increasing flux through Und-P pathways is an effective way to increase Und-P levels in different strain backgrounds.

### Increasing Und-P availability increases endogenous glycan expression

**Fig. 2:**
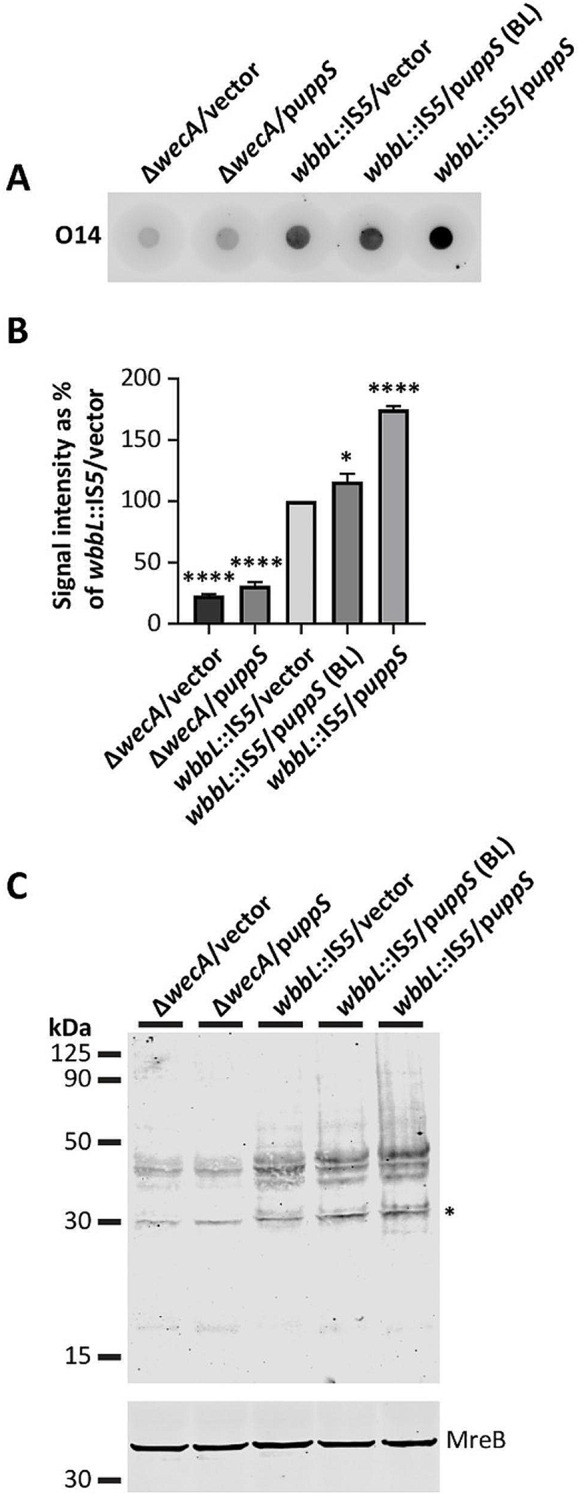
Increasing Und-P levels promotes enterobacterial common antigen surface expression in *E*. *coli*. (**A**) Dot blot showing that increasing Und-P levels by overexpressing *uppS* promotes surface expression of ECA in *E*. *coli*. Cells were grown at 37 °C in LB media containing 500 µM IPTG (0 µM IPTG for baseline [BL]). Cells lacking WecA do not produce ECA (negative control). (**B**) Densitometry was performed from dot blots to quantitate surface expression of ECA. Signal values are given as a percent of the average *wbbL*::IS*5*/vector (*wbbL*::IS*5* is *E*. *coli* MG1655) signal. All data are representative of three independent experiments. Error bars show +/- standard error of the means. Significance was determined by using an ordinary one-way ANOVA test with Dunnett’s multiple correction. Asterisks above sample bars denote significance relative to *wbbL*::IS*5*/vector. *p < 0.05, **** <0.0001. (**C**) Western blotting was used to examine the effect of increasing Und-P levels on ECA chain length from cells grown in panel A. *, nonspecific band. MreB, which has a predicted molecular mass of 37 kDa, served as the loading control. The *E*. *coli* strains shown are MAJ286 (*wbbL*::IS*5*/vector), MAJ981 (Δ*wecA*/vector), MAJ1354 (*wbbL*::IS*5*/p*uppS*), and MAJ1677 (Δ*wecA*/p*uppS*).

Several studies have shown that *uppS* overexpression (increases Und-P levels) increases Und-PP-linked intermediate formation in *E*. *coli* [18, 20, 21]. However, to our knowledge, no study has clearly linked increased Und-P to the production of finished glycans. Therefore, to determine if increasing Und-P levels increases production of finished glycans, we measured the effect of *uppS* overexpression on ECA (O14 antigen) surface expression in *E*. *coli wbbL*::IS*5* (MG1655) cells. We note that *wbbL*::IS*5* cells express two forms of ECA at the cell surface, (1) LPS-linked ECA (due to the loss of O-antigen expression [40]) and (2) phosphoglyceride-linked ECA (a third cyclic form is also expressed in the periplasm) [41]. Interestingly, dot blot analysis revealed that *wbbL*::IS*5*/p*uppS* cells produce 75% more signal at the cell surface than *wbbL*::IS*5*/vector cells (Fig. 2A and B); cells lacking WecA did not produce an appreciable amount of signal, even when induced to overexpress *uppS* (Fig. 2A and B). Western blot analysis revealed similar increases in ECA expression, with the strongest effects observed for ECA chains less than 50 kDa (Fig. 2C, compare *wbbL*::IS*5*/vector to *wbbL*::IS*5*/p*uppS*). Bands corresponding to longer ECA chains (> 50 kDa) were also stronger in cells overexpressing *uppS* (Fig. 2C). We also observed a non-specific band ~ 30 kDa whose expression varied in our strains (Fig. 2C). We are currently investigating the source of this band. In short, our results indicate that increasing Und-P availability increases endogenous glycan expression in bacteria.

### Increasing Und-P availability increases recombinant glycan expression

Since increasing Und-P availability increased endogenous glycan expression (Fig. 2), we next sought to determine if similar effects would occur for *E*. *coli* cells expressing a non-native recombinant glycan. For these studies, we examined the effect of increasing Und-P availability in *E*. *coli* cells expressing the capsular polysaccharide from *Streptococcus pneumoniae* serotype 4 (SP4); SP4 was expressed from an IPTG-inducible promoter on plasmid pB4 [29]. To do this, we first examined the effect of increasing Und-P on SP4 expression in *E*. *coli* W3110. To our surprise, Western blot analysis revealed that *uppS* overexpression produced little effect on SP4 expression in W3110 (Fig. S2). However, since *N*-acetylgalactosamine (GalNAc) availability limits recombinant SP4 expression [29], we overexpressed *uppS* in *E*. *coli* Falcon cells, a derivative of W3110 that expresses an integrated copy of the *gne* epimerase from *Campylobacter jejuni* at the *wecA* locus (Δ*wecA*-*wzzE*[*gne*]) [29, 42]. Gne is a bifunctional UDP-GlcNAc/Glc 4-epimerase that increases GalNAc availability by converting UDP-GlcNAc to UDP-GalNAc [42]. Subsequent analysis of this strain by Western blot revealed that *E*. *coli* Falcon cells express moderately more SP4 than W3110 cells (Fig. S2, compare W3110/pB4/vector to Falcon/pB4/vector) and the addition of *uppS* overexpression further increased SP4 expression in this strain background (Fig. S2, compare Falcon/pB4/vector to Falcon/pB4/p*uppS*).

**Fig. 3:**
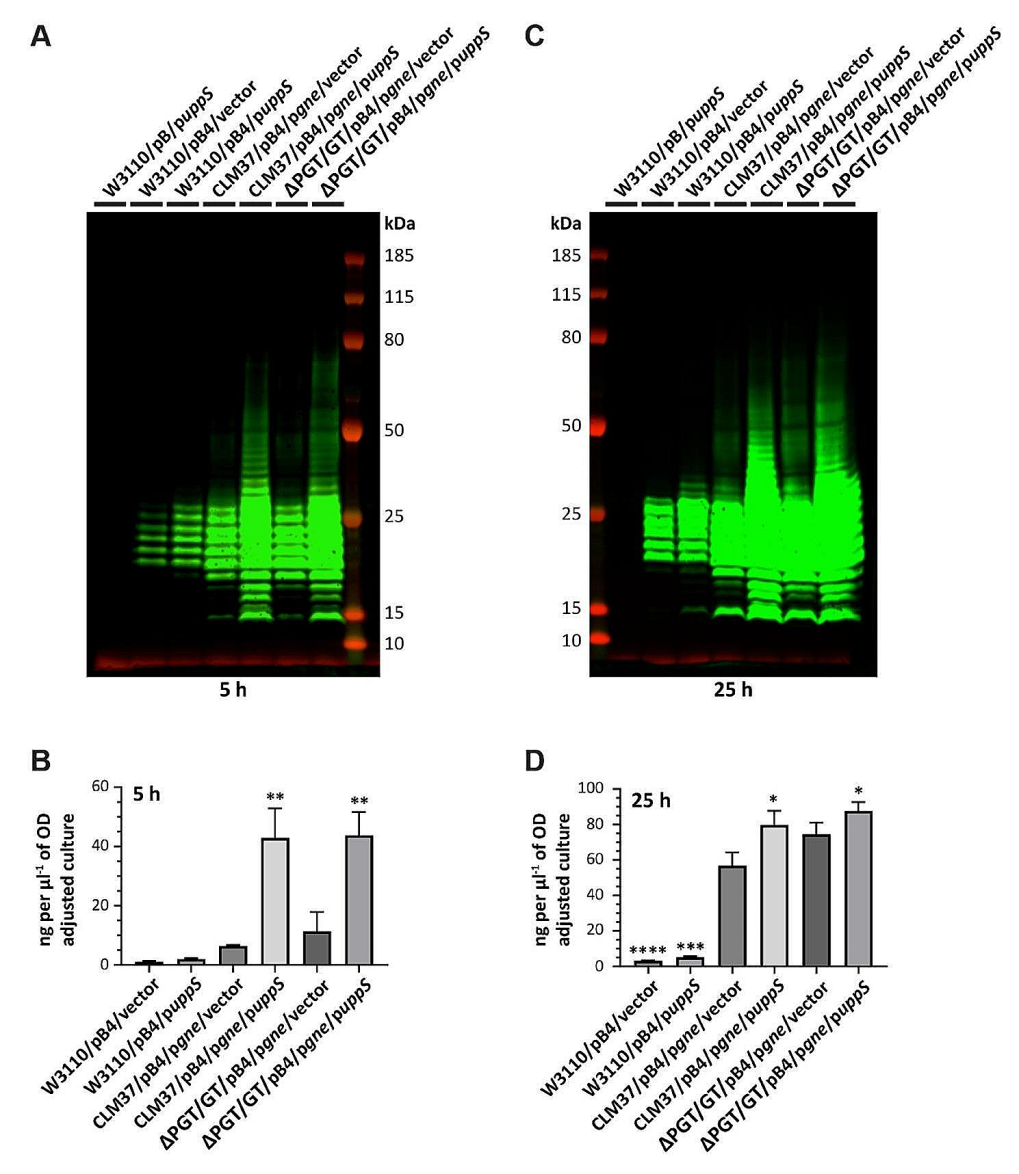
Increasing Und-P levels promotes recombinantly expressed *S*. *pneumoniae* capsular polysaccharide in *E*. *coli*. (**A** and **C**) Western blots showing that increasing Und-P levels enhances production of *S*. *pneumoniae* capsule serotype 4 in *E*. *coli* cells harboring plasmid pB4 (contains capsule loci). Cells with the indicated genotypes were grown at 28 °C in 2YP media for 5 or 25 h. Lysed, whole cell samples were then separated by SDS-PAGE on a 4–12% bis-tris gel and detected using anti-serotype CPS primary and anti-rabbit fluorescent secondary antibody. Results are representative of three independent experiments. (**B** and **D**) ELISA. Capsular polysaccharide quantitated from cells grown in panels A and C. All experiments represent three biological replicates, with each sample probed in duplicate. Values are expressed as means of ng capsular polysaccharide serotype 4 production per µl of culture adjusted to OD_600_ = 10. Error bars show standard errors of the means. Significance was determined using an ordinary one-way ANOVA with Dunnett’s multiple correction. Asterisks above sample bars denote significance relative to CLM37/pB4/p*gne*/vector. *p < 0.05, **<0.005, ***<0.001, **** <0.0001. Absolute capsular polysaccharide values are detailed in Table 1. The *E*. *coli* strains shown are EJK1 (W3110/pB/p*uppS*), EJK2 (W3110/pB4/vector), EJK3 (W3110/pB4/p*uppS*), EJK7 (CLM37/pB4/p*gne*/vector), EJK8 (CLM37/pB4/p*gne*/p*uppS*), EJK11 (ΔPGT/GT/pB4/p*gne*/vector), and EJK12 (ΔPGT/GT/pB4/p*gne*/p*uppS*).

Since Gne activity appeared to be limiting in our strain backgrounds, we switched to overexpressing *gne* from a plasmid (p*gne*) in *E*. *coli* CLM37 cells, a derivative of W3110 lacking WecA [43]. Subsequent analysis by Western blot revealed that *gne* overexpression increased SP4 expression in CLM37 cells, particularly for SP4 chains < 30 kDa (Fig. 3A and C). Quantitation of SP4 levels by ELISA showed that *gne* overexpression in CLM37/pB4/vector cells increased SP4 expression 522% after 5 h induction and 1687% after 25 h when compared to the typical output of our previous expression efforts (Fig. 3B and D; Table 1, compare W3110/pB4/vector to CLM37/pB4/p*gne*/vector cells). Notably, the addition of *uppS* overexpression in CLM37/pB4/p*gne* cells further increased SP4 expression in this strain background; CLM37/pB4/p*gne*/p*uppS* cells expressed significantly more SP4 chains that were also longer on average after 5- and 25-hours induction (Fig. 3A and C, compare CLM37/pB4/p*gne*/vector to CLM37/pB4/p*gne*/p*uppS*). Quantitation of SP4 by ELISA showed that *uppS* overexpression in CLM37/pB4/p*gne* cells increased SP4 expression 568% after 5 h induction and 40% after 25 h (Fig. 3B and D; Table 1). We next investigated SP4 expression in our Und-P pathway minimized strain. Western blot and ELISA analysis revealed that *gne* overexpression in ΔPGT/GT cells mirrored results obtained for CLM37 cells (Fig. 3, compare CLM37/pB4/p*gne*/vector to ΔPGT/GT/pB4/p*gne*/vector). Similar trends were also observed for the addition of p*uppS* into ΔPGT/GT/pB4/p*gne* cells, noting that SP4 expression was highest in this strain background after 5- and 25-hours induction (Fig. 3; Table 1, compare CLM37/pB4/p*gne*/p*uppS* to ΔPGT/GT/pB4/p*gne*/p*uppS*).

Altogether, these data confirm that increasing the free pool of Und-P serves to increase glycan expression. Since polysaccharides like SP4 are used as antigens in polysaccharide-based vaccines [44] and because glycan length is correlated with immunogenicity [45], our findings have important implications for the expression and production of glycans in *E*. *coli* cells that may be used for a variety of therapeutic purposes.

**Table 1:** Quantification of SP4 production by sandwich ELISA

ng SP4 polysaccharide per ul^− 1^ of OD_600_ adjusted culture at 5 h								
Strain^a^	Plasmid			Replicate			Average	Deviation
	pB4	p*gne*	p*uppS*^b^	**1**	**2**	**3**		
W3110	+	-	v	0.60	1.56	0.93	1.03	0.49
	+	-	+	1.58	2.57	1.91	2.02	0.50
CLM37	+	+	v	5.74	6.78	6.72	6.41	0.58
	+	+	+	24.74	59.46	44.34	42.85	17.41
ΔPGT/GT	+	+	v	10.54	23.06	0.50	11.36	11.30
	+	+	+	47.82	54.83	28.58	43.74	13.60
**ng SP4 polysaccharide per ul** ^**− 1**^ **of OD**_**600**_ **adjusted culture at 25 h**								
W3110	+	-	v	2.80	3.73	3.02	3.18	0.49
	+	-	+	4.19	6.38	4.98	5.18	1.11
CLM37	+	+	v	44.81	70.26	55.40	56.83	12.79
	+	+	+	63.95	88.22	87.23	79.80	13.74
ΔPGT/GT	+	+	v	62.48	84.84	76.33	74.55	11.28
	+	+	+	79.98	97.34	85.35	87.56	8.89

^a^*E*. *coli* strains: EJK2 (W3110/pB4/vector), EJK3 (W3110/pB4/p*uppS*), EJK7 (CLM37/pB4/p*gne*/vector), EJK8 (CLM37/pB4/p*gne*/p*uppS*), EJK11 (ΔPGT/GT/pB4/p*gne*/vector), and EJK12 (ΔPGT/GT/pB4/p*gne*/p*uppS*)

^b^Plasmids: v, vector; +, p*uppS*

### Increasing Und-P availability increases glycan expression in bacteria used to make glycoproteins

**Fig. 4:**
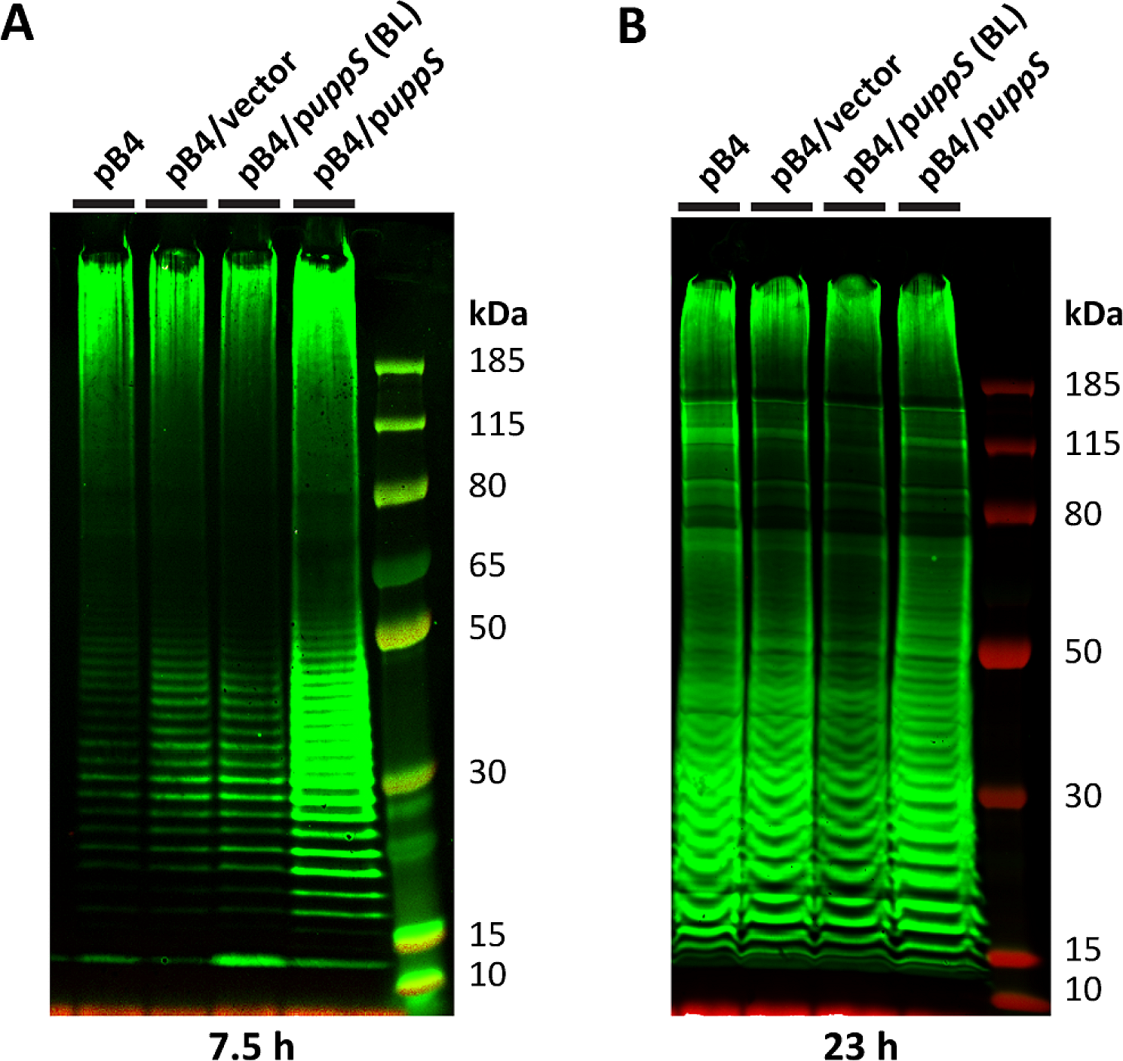
Increasing Und-P levels increases glycan expression in *E*. *coli* cells engineered for enhanced expression of *S*. *pneumoniae* capsular polysaccharides. Western blots showing that increasing Und-P levels by overexpressing *uppS* enhances expression of *S*. *pneumoniae* capsule serotype 4 (expressed from pB4) in *E*. *coli* Sparrowhawk cells over shorter periods of time. Cells were grown at 28 °C in 2YP media containing 500 µM IPTG (0 µM IPTG for baseline [BL]) for 7.5 h (panel **A**) or 23 h (panel **B**). Lysed, whole cell samples were then separated by SDS-PAGE on a 4–12% bis-tris gel and detected using anti-serotype CPS primary and anti-rabbit fluorescent secondary antibody. The *E*. *coli* strains shown are EJK13 (pB4), EJK14 (pB4/vector), and EJK15 (pB4/p*uppS*).

*E*. *coli* strains are used to produce glycoprotein vaccine candidates by a recombinant process termed Protein Glycan Coupling Technology (PGCT). PGCT initiates with the assembly of sugar monomers (like GalNAc) onto Und-P and culminates with the transfer of oligosaccharides onto immunogenic carrier proteins (in the periplasmic space) by enzymes possessing oligosaccharyltransferase activity like *Campylobacter jejuni* PglB [46]. Since Und-P availability is central to PGCT, we reasoned that increasing Und-P could be used to improve recombinant glycan expression in PGCT. As a first step to address this question, we expressed SP4 in *E*. *coli* Hobby cells [29], a derivative of Falcon deleted for the O-antigen WaaL ligase, which prevents SP4 from being transferred onto lipid A-core. Curiously, SP4 expression was poor in Hobby cells and *uppS* overexpression produced no observable effect (Fig. S2). Since plasmid pB4 does not contain the *wzDE* chain regulators from the *S*. *pneumoniae* capsule locus [28], this suggested that reintroducing *wzDE* may produce an effect similar to *gne* and *uppS* co-overexpression. Thus, *uppS* was overexpressed in *E*. *coli* Sparrowhawk cells, a derivative of Hobby cells that expresses *wzDE* from the *wzzB* locus (Δ*wzzB*[*wzD*-*wzE*]) [29]. As shown in the Western blot in Fig. 4A, increasing Und-P strongly increased SP4 expression in Sparrowhawk cells after 7.5 h induction, with the strongest effects observed for SP4 chains less than 50 kDa. In retrospect, these results suggest that Und-PP-linked SP4 intermediates were accumulating (but not polymerizing efficiently) in Falcon and Hobby cells overexpressing *uppS* (Fig. S2). We also examined SP4 expression in Sparrowhawk cells after overnight induction. However, increasing Und-P did not appear to improve SP4 expression at this time point (Fig. 4B). Collectively, these data confirm that increasing Und-P availability can be used to increase recombinant glycan expression, especially over shorter periods of time. Current efforts are focused on engineering Und-P pathways to improve other aspects of PGCT systems.

## Discussion

**Fig. 5:**
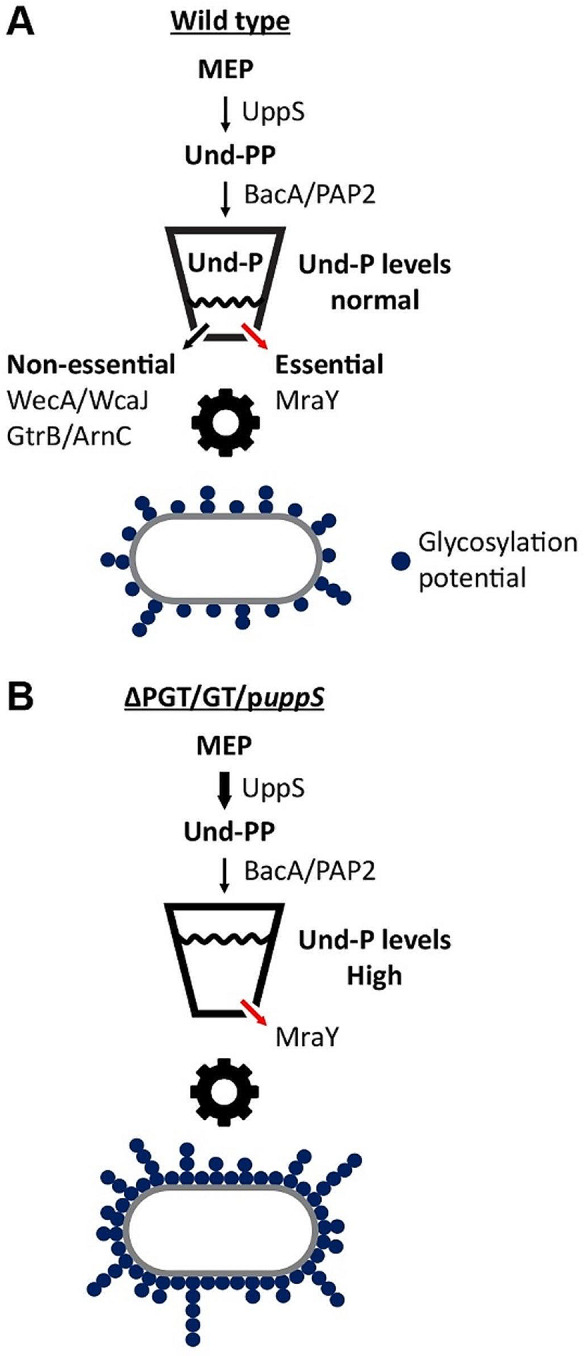
Model depicting the effect of increasing Und-P on glycan expression. (**A**) Und-P synthesis. Und-P is synthesized via the methylerythritol phosphate (MEP) pathway. Und-P is first synthesized as Und-PP by the UppS synthase. Integral membrane phosphatases (BacA/PAP2 family proteins) then dephosphorylate Und-PP to Und-P (the level of which is visually indicated by the black wavy line in the bucket icon). Und-P is then distributed to phosphoglycosyl transferases and glycosyltransferases (PGT/GTs) that prime glycan assembly in non-essential and essential (peptidoglycan) pathways. In *E*. *coli* K-strains, PGT/GTs transfer precursors of peptidoglycan (MraY), O-antigen (WecA), enterobacterial common antigen (WecA), and colanic acid (WcaJ) to Und-P. Und-P is also required to glycosylate lipid A with aminoarabinose (ArnC) and O-antigen with glucose (GtrB). Since Und-P limits glycan assembly (gear icon), the glycosylation potential of any bacterium is limited by Und-P availability. (**B**) Simultaneously deleting all non-essential PGT/GTs and overexpressing *uppS* increases the amount of Und-P available for recombinant glycan expression. Our findings indicate that increasing Und-P levels increases both glycan yield and chain length

Many of the sugars that coat and protect bacteria are produced from oligosaccharide precursors that are built on an essential lipid carrier known as undecaprenyl phosphate (Und-P). While most research has focused on establishing the role of Und-P in scaffolding glycan synthesis, as well as understanding the physiological consequences of producing too little Und-P, less is known about the effects of increasing Und-P on glycan assembly. The present results demonstrate that increasing Und-P levels potentiates expression of native and foreign glycans. Since Und-P is a universal lipid carrier, we argue that similar results will occur for potentially any Und-P-dependent polymer expressed in *E*. *coli* cells (i.e., a rising tide lifts all boats) (Fig. 5). While increasing Und-P levels was well-tolerated in *E*. *coli*, whether similar effects occur in other bacteria will need to be determined. In summary, Und-P availability forms a critical bottleneck that can be exploited to improve glycan expression in the biotechnology benchmark bacterium *E*. *coli*.

### The connection between Und-P and glycan chain length

Bacterial polysaccharides like SP4 trigger antibody production by cross-linking B cell receptors on the surface of naïve B cells [47]. For this reason, the ability of plain polysaccharide vaccines to elicit effective immune responses is highly correlated with glycan chain length (i.e., longer glycan chains elicit stronger immune responses) [45]. To that end, there are likely many factors that influence glycan chain length. For example, in Wzx/Wzy-dependent systems (like SP4), Wzx translocase activity limits Und-PP-linked intermediate availability and, consequently, Wzy polymerase activity [48, 49]. Similarly, the amount of Wzy polymerases [50] and the activity of chain-length regulatory Wzz proteins [51] are known to affect glycan chain length. Our results also show that the availability of chain length regulators (WzDE, Fig. 4), as well as sugars (GalNAc, Fig. 3A and C), play important roles in determining SP4 chain length. In addition, cells that contain more Und-P also produce more glycans that are also increased in length (Fig. 3A and C). More work will be needed to determine the molecular basis for this connection, including determining the impact of Und-P levels on Wzx/Wzy activity, as well as growth dynamics and membrane composition. Since Und-P is a universal lipid carrier, the connection between Und-P and glycan chain length could be leveraged in pathogens like *S*. *pneumoniae*, whose capsular polysaccharide forms the protective basis of the pneumococcal glycoconjugate vaccine [44].

### Revisiting BacA and Und-PP dephosphorylation

Und-P is central to glycan expression technology, yet most strategies to produce glycans in bacteria do not address the limiting nature of Und-P. Why? The answer to this question is likely rooted in the discovery of BacA, which dephosphorylates Und-PP to Und-P. BacA was originally identified in a screen for genes whose overexpression confers resistance to bacitracin [52], an antibiotic that binds the pyrophosphate group on Und-PP [53]. Since BacA also binds Und-PP, an excess of BacA is expected to prevent Und-PP sequestration by outcompeting bacitracin for Und-PP. However, the prevailing hypothesis at the time of its discovery was that BacA mediated resistance by increasing the free pool of Und-P. While *bacA* overexpression is expected to increase the rate of Und-PP dephosphorylation, no study has shown that increasing BacA activity increases the free pool of Und-P. On the contrary, *bacA* overexpression does not reverse the effects of a low Und-P state [33, 54] nor does it improve glycosylation efficiency [27]. Thus, early assumptions about *bacA* led to negative results that otherwise discouraged research into Und-P by glycan expression systems. For this reason, conclusions based on *bacA* overexpression should be reevaluated, ideally by overexpressing *uppS* or earlier steps in Und-P(P) synthesis.

### Defining how much Und-P is too much

Polyprenyl phosphates like Und-P are prone to form micelles and disrupt membrane architecture when present in high concentrations [19]. For this reason, there is probably a limit to the amount of free (i.e., unbound) Und-P that cells can stably maintain. However, in addition to its free form, Und-P can also exist in a bound form as a Und-P- or Und-PP-linked intermediate. Interestingly, several recent studies have shown that cells can accommodate large quantities of Und-PP-linked intermediates [18, 20, 21]. Thus, one way to maximize the pool of Und-P without crossing the threshold for free Und-P is to steadily increase the pool of Und-P while simultaneously synthesizing more Und-PP-linked intermediates. That said, we note that the accumulation of Und-PP-linked intermediates can be deleterious (due to Und-P sequestration) [33, 55, 56], even in systems engineered to contain more Und-P [57].

### Reallocating Und-P from essential pathways

Presumably, the only pathways utilizing Und-P in ΔPGT/GT/pB4 derivatives are SP4 and cell wall synthesis. In a situation where the free pool of Und-P reaches total occupancy in this strain background, SP4 production is maxed out. At this point, one way to liberate additional Und-P for SP4 production is to reduce the flux of Und-P to cell wall synthesis. Since the cell wall is essential, this brings up an interesting question: how much cell wall synthesis is necessary? The answer to this question is rooted in the amount of cell wall material required to maintain growth and integrity. Based on studies using *E*. *coli* strains auxotrophic for diaminopimelic acid (i.e., an essential component for cell wall synthesis), a two-fold reduction in the amount of cell wall per unit of cell surface area is compatible with normal growth and morphology in this bacterium [58, 59]. Thus, bacteria like *E*. *coli* produce an excess of cell wall material, presumably as a fail-safe against disruptions in cell wall synthesis. This observation suggests that cell wall synthesis can be reduced to increase Und-P levels in ΔPGT/GT cells.

## Conclusions

Bacteria assemble glycans on an essential lipid carrier known as undecaprenyl phosphate (Und-P). Normally, the amount of Und-P available for glycan expression in bacteria is limited by competition between different Und-P-utilizing pathways and by the overall rate of Und-P synthesis. Here, we show that decreasing competition for Und-P and increasing Und-P synthesis promote Und-P availability and, consequently, Und-P-dependent polymer formation. Collectively, our findings show that Und-P levels should be a prime consideration for glycoengineering, which has far-reaching implications. The rational engineering of bacteria to produce greater quantities of Und-P will increase the yield of glycan expression, thereby making glycan-based therapies more affordable.

## Materials and methods

### General procedures

All strains, plasmids, and primers are listed in Tables S2, S3, and S4, respectively. Cells were cultured in LB miller broth (1% tryptone, 0.5% yeast extract, and 1% NaCl), 2YP broth (2% yeast extract and 4% peptone), or modified TB media (2.4% yeast extract, 2.0% tryptone, 0.5% glycerol, 100 mM phosphate pH 7.4, and 2 mM MgCl_2_). Plates contained 1.5% agar. As required, antibiotics were used at the following concentrations: 100 µg ml^− 1^ ampicillin, 50 µg ml^− 1^ kanamycin, 20 µg ml^− 1^ tetracycline, and 80 µg ml^− 1^ spectinomycin.

### Strain construction

*E*. *coli* MG1655 *wbbL* + is the parent strain for this study and expresses O-antigen, which obscures the P1 binding receptor (i.e., LPS core) [60]. Thus, all gene deletions were constructed by using lambda Red recombination. Kanamycin and chloramphenicol resistance markers were excised by using FLP recombinase produced on pCP20 [61]. All gene deletions were verified by PCR. Sequences to generate deletion and check primers were obtained from the Ecocyc database [62]. PCR fragments and plasmids were purified by using kits obtained from Qiagen.

### Morphological analyses of Und-P pathway mutants

Overnight cultures were diluted 1:2,000 in TB medium (Und-P pathway mutants) or TB medium containing ampicillin and 500 µM IPTG (ΔPGT/GT derivatives) and grown at 37 °C to an optical density at 600 (OD_600_) of 0.4–0.6 (approximately 10 doublings). Live cells were then spotted onto 1% agarose pads and imaged by phase-contrast microscopy by using an XM10 monochrome camera coupled to an Olympus BX60 microscope. Live cells for flow cytometry were prepared by pelleting 1 mL of cells (above), washing twice in phosphate-buffered saline (PBS), and diluting to an OD_600_ ~ 0.05. Cells were then analyzed by using the forward scatter detector in a BD LSRFortessa Flow Cytometer at the UAMS Flow Cytometry Core Facility.

### Preparing cell lysates for Und-P quantification

Overnight cultures were diluted 1:2,000 in 10 mL TB (Und-P pathway mutants) or TB containing 500 µM IPTG (ΔPGT/GT derivatives) and incubated at 37 °C for 3.5 h or 24 h, respectively. Cells were then pelleted, washed with 0.9% NaCl, and resuspended in 0.7 ml of water. In parallel, cells were plated on LB medium to obtain the number of colony forming units per ml (cfu ml^− 1^). Cells were then lysed by transferring the suspension to glass tubes containing 3 ml of a 2:1 methanol:chloroform mixture and incubating at room temperature for 20 min. Next, glass tubes were placed in a CentriVap and centrifuged without vacuum for 20 min, after which the soluble supernatant was transferred to new glass tubes and placed at -80 °C. Once a slurry formed, the glass tubes were placed back in the CentriVap with vacuum and dried to completion. The crude cell lysate was stored in 0.2 ml of n-propanol and 0.1% ammonium hydroxide (1:3) at − 20 °C for up to one week.

### LC-MS conditions for Und-P quantitation

Cell lysates (see above) were centrifuged at 10,000 x *g* briefly and then 5 µL were injected for LC-MS analysis. Samples were analyzed on an Agilent 1260 Infinity II system equipped with a single quadrupole electrospray ionization (ESI) MS detector. A high-pH stable Waters Xbridge Peptide BEH C18 column (4.6 × 50 mm with 2.5 µM particle size) was used. The mobile phases used included 0.1% Ammonium Hydroxide (A) and n-propanol (B). A gradient was used to evaluate cell lysates containing Und-P starting at 15% B, which was increased to 95% over 10.0 min at 1 mL min^− 1^, then held at 95% B for 2 min, then decreased to 15% B for an additional 2 min. A 4 min post-run at 15% B was run in-between injections. The ESI capillary voltage was applied to 4,000 V with a drying gas temperature of 350 °C at 12 L min^− 1^. Scanning ion mode was set to detect the [M-1 H]-1 ion species of Und-P (845.7 m/z) with a peak width of 0.1 min. A fragmentor voltage of 240 V was found to be optimal for Und-P detection.

### Und-P standard curve generation and quantitation analysis

A two-step chemoenzymatic approach was employed to prepare Und-P as described previously [63]. To quantify the concentration of the sample, total phosphorous content was determined and compared to a standard curve [64, 65]. Und-P (100 µM) was supplemented to an aliquot of wild type cell lysate and the AUC_Total_ was determined by using the ChemStation integration tool (Agilent Technologies). Endogenous background Und-P, AUC_Endo_, was subtracted from AUC_Total_ to yield AUC_Adjusted_ to reduce the influence of the sample matrix effects. Injections of 25, 50, 100, 500, and 1,000 pmol of Und-P were used to generate the LC-MS response curve for quantitation. For cell lysate quantitation, the slope and y-intercept from the Und-P standard were used to calculate the quantity of Und-P based on AUC. Und-P levels were normalized by dividing Und-P measurements by the mean cfu ml^− 1^.

### ECA dot blot

Cells were cultured in LB medium containing ampicillin and 500 µM IPTG (no IPTG was added to p*uppS* baseline [BL]) at 37 °C overnight. Total cell material was matched by centrifuging the equivalent of 1 ml of culture at an OD_600_ = 1.0 and resuspending the pellet in 20 µl of PBS. 3 µl aliquots were then applied to a nitrocellulose membrane and allowed to dry for 20 min. Membranes were blocked with 5% skimmed milk in PBS for 1 h. Rabbit anti-ECA serum (Statens Serum Institut) was then applied at 1:1,000 in PBS for 3 h. The primary antibody was detected by using the goat anti-rabbit IgG conjugated to Alexa Fluor 488 (Thermo Fisher Scientific) secondary antibody and was applied at 1:4,000 for 1 h. Blots were imaged by using a ChemiDoc MP imaging system (Bio-Rad Laboratories). Signals corresponding to surface ECA were quantified by using ImageJ.

### ECA immunoblot

Cells were cultured in LB media containing 500 µM IPTG (0 µM IPTG was added to p*uppS* baseline [BL]) at 37 °C overnight. Total cell material was matched by centrifuging the equivalent of 1 ml of culture at an OD_600_ = 1.0 and resuspending the pellet in 25 µL BugBuster® Protein Extraction Reagent (MilliporeSigma) containing 1:100 benzonase. Cell suspensions were incubated at room temperature for 10 min, mixed with loading buffer (LI-COR Biosciences), and boiled for 5 min. 10 µL aliquots were then separated on 12% Mini-PROTEAN® TGX™ (Bio-Rad Laboratories) gels. ECA polymers were transferred onto nitrocellulose membranes using a Trans Blot Turbo system (Bio-Rad Laboratories) set at 25 V (1.3 amps) for 7 min. Membranes were dried at 37 °C and then blocked with 5% skimmed milk in PBS for 1 h. Membranes were then washed three times with PBS (0.2% Tween 20). Rabbit anti-ECA antibody (a gift from Angela Mitchell) was then applied at 1:24,000 in PBS (0.2% Tween 20) for 1 h. The membranes were then washed three times with PBS (0.2% Tween 20). The primary antibody was detected by using the goat anti-rabbit IgG conjugated to IRDye680 (LI-COR Biosciences) secondary antibody at 1:10,000 for 1 h. Membranes were washed a further three times in PBS (0.2% Tween 20) before signal detection with an Odyssey LI-COR detection system (LI-COR Biosciences). Similar procedures were used to detect MreB; the primary rabbit anti-MreB antibody was applied at 1:2,000 for 1 h and the secondary goat anti-rabbit IgG conjugated to AF488 (Thermo Fisher Scientific) antibody at 1:4,000 for 1 h.

### Recombinant capsule expression and immunoblot

Overnight cultures were diluted to an OD_600_ of 0.03 in 10 ml of 2YP medium containing 500 µM of IPTG and cultured at 28 °C. Cells from 1.5 ml of culture grown from various times were withdrawn and pelleted. Pellets were then resuspended to an OD_600_ = 10.0 in PBS, mixed with 1 mg ml^− 1^ lysozyme and 40 U ml^− 1^ benzonase, and boiled for 10 min. Lysed samples were mixed with NuPAGE LDS sample buffer (Invitrogen) and separated on Bolt 4–12% Bis-Tris 1 mm gels in MOPS buffer (Invitrogen) at 100 V for 2 h 25 min in an ice bath. Capsular polysaccharides were transferred onto nitrocellulose membranes using an iBlot 2 dry transfer system (Thermo Fisher) set at 20 V for 1 min, 23 V for 4 min, and 25 V for 2 min. Membranes were blocked with 2% skimmed milk in PBS for 1 h. *S. pneumoniae* Serotype 4 (SP4) rabbit anti-capsule antibody (Statens Serum Institut) was then applied at 1:1,000 in PBS containing 2% skimmed milk and 0.1% Tween 20 for 1 h. The membranes were then washed three times with PBS (0.1% Tween 20). The primary antibody was detected by using the goat anti-rabbit IgG conjugated to IRDye800 (LI-COR Biosciences) secondary antibody at 1:10,000 for 45 min. Membranes were washed a further three times in PBS (0.1% Tween 20) and once with PBS before signal detection with an Odyssey LI-COR detection system (LI-COR Biosciences).

### Indirect enzyme-linked immunosorbent assay (ELISA)

Dilutions of boiled *E*. *coli* lysates were used to coat a MaxiSorp microtiter plate (Nunc) overnight at 4 °C. A standard curve was generated by using dilutions of purified type 4 pneumococcal polysaccharide (Statens Serum Institut). Wells were then washed with PBS containing 0.05% Tween 20 four times, blocked with PBS containing 5% skimmed milk for 1.5 h, and washed twice again. SP4 rabbit anti-capsule antibody (Statens Serum Institut, Denmark) was then applied at 1:1,000 in PBS containing 2% skimmed milk for 1 h. The wells were then washed four times with PBS 0.05% Tween 20. The primary antibody was detected by using the goat anti-rabbit IgG HRP (Abcam) secondary antibody at 1:20,000 in PBS containing 2% milk for 45 min. After washing four times with PBS 0.05% Tween 20, tetramethylbenzidine (eBioscience) was added, and the reaction was stopped with 2M H_2_SO_4_ (sulfuric acid). Indirect ELISA detection was performed using a SpectrMax iD5 microplate reader (Molecular Devices) at an absorbance of 450 nm.

### Electronic supplementary material

Below is the link to the electronic supplementary material.

Supplementary Material 1

## Data Availability

No datasets were generated or analysed during the current study.
