# Supplementary material for: Engineering Escherichia coli for increased Und-P availability leads to material improvements in glycan expression technology
Source: Microb Cell Fact. 2024 Mar 1;23:72. doi: 10.1186/s12934-024-02339-8 (PMC10908060; doi:10.1186/s12934-024-02339-8)
Supplement: Supplementary file 1 — Supplementary Material 1 [file 12934_2024_2339_MOESM1_ESM.pdf]

Supplemental information for:

Engineering *Escherichia coli* for increased Und-P availability leads to material improvements in glycan expression technology

Emily J. Kay[1], Manoj K. Dooda[2], Joseph C. Bryant[3], Amanda J. Reid[4], Brendan W. Wren[1], Jerry M. Troutman[4,5], and Matthew A. Jorgenson[3]*

1. Department of Infection Biology, London School of Hygiene and Tropical Medicine, London WC1E 7HT, UK
2. Department of Biological Sciences, University of North Carolina at Charlotte, Charlotte, NC 28223, USA
3. Department of Microbiology and Immunology, University of Arkansas for Medical Sciences, Little Rock, AR 72205, USA
4. Nanoscale Science Program, University of North Carolina at Charlotte, Charlotte, NC 28223, USA
5. Department of Chemistry, University of North Carolina at Charlotte, Charlotte, NC 28223, USA

*Corresponding author

4301 West Markham St. / Biomed I, Room 511 / Little Rock, AR 72205

E-mail: majorgenson@uams.edu

Phone: 501-686-7706

Table S1. Intracellular Quantitation of Und-P by RP-LC-MS

Molecules of Und-P per cfu^{-1} in Und-P pathway mutants					
**Strain[a]**	**Replicate 1**	**Replicate 2**	**Replicate 3**	**Average**	**Deviation**
Wild type	$1.29x10^5$	$6.62x10^4$	$1.73x10^5$	$1.23x10^5$	$5.38x10^4$
Δ*gtrB*	$6.52x10^4$	$6.37x10^4$	$1.22x10^5$	$8.38x10^4$	$3.34x10^4$
Δ*wecB*	$1.16x10^5$	$9.38x10^4$	$8.12x10^4$	$9.71x10^4$	$1.77x10^4$
Δ*arnC*	$1.37x10^5$	$1.11x10^5$	$1.17x10^5$	$1.22x10^5$	$1.36x10^4$
wbbL::IS*5*	$9.49x10^4$	$2.27x10^5$	$6.35x10^4$	$1.29x10^5$	$8.70x10^4$
Δ*wecA*	$1.18x10^5$	$1.18x10^5$	$1.68x10^5$	$1.35x10^5$	$2.89x10^4$
Δ*wcaJ*	$1.01x10^5$	$1.13x10^5$	$2.70x10^5$	$1.64x10^5$	$9.98x10^4$
ΔPGT/GT	$4.10x10^5$	$2.05x10^5$	$2.89x10^5$	$3.01x10^5$	$1.03x10^5$
**Molecules of Und-P per cfu^{-1} in strains harboring plasmids**					
ΔPGT/GT/vector	$7.98x10^4$	$8.25x10^4$	$9.27x10^4$	$8.50x10^4$	$6.80x10^4$
ΔPGT/GT/p*uppS*	$5.45x10^5$	$3.36x10^5$	$2.80x10^5$	$3.87x10^5$	$1.39x10^5$

[a]Strains: MAJ330 (wild type), MAJ484 (Δ*gtrB*), MAJ362 (Δ*wecB*), MAJ1067 (Δ*arnC*), MAJ1 (*wbbL*::IS*5*), MAJ347 (Δ*wecA*), MAJ1068 (Δ*wcaJ*), MAJ557 (ΔPGT/GT), MAJ1385 (ΔPGT/GT/vector), and MAJ1386 (ΔPGT/GT/p*uppS*).

Table S2. Strains used in this study

Strain	Relevant features	Source or reference
CLM37	W3110 Δ*wecA*::*frt*	[1]
W3110	*rph*-1 *IN*(*rrnD-rrnE*)*1 wbbL*::IS*5*	Lab collection
Sparrowhawk	W3110 Δ*lpxM* Δ*wecA*-*wzzE*(*gne*) Δ*waaL* Δ*wzzB*(*wzD-wzE*)	[2]
Falcon	W3110 Δ*lpxM* Δ*wecA*-*wzzE*(*gne*)	[2]
Hobby	W3110 Δ*lpxM* Δ*wecA*-*wzzE*(*gne*) Δ*waaL*	[2]
EJK1	W3110/pB/p*uppS*	This study
EJK2	W3110/pB4/pDSW204	This study
EJK3	W3110/pB4/pMAJ9	This study
EJK7	CLM37/pB4/p*gne/*pDSW204	This study
EJK8	CLM37/pB4/p*gne*/pMAJ9	This study
EJK11	MAJ557/pB4/p*gne*/pDSW204	This study
EJK12	MAJ557/pB4/p*gne*/pMAJ9	This study
EJK13	Sparrowhawk/pB4	[2]
EJK14	Sparrowhawk/pB4/pDSW204	This study
EJK15	Sparrowhawk/pB4/pMAJ9	This study
EJK16	Falcon/pB4/pDSW204	This study
EJK17	Falcon/pB4/pMAJ9	This study
EJK18	Hobby/pB4/pDSW204	This study
EJK19	Hobby/pB4/pMAJ9	This study
EJK20	W3110/pB	This study
MAJ1	MG1655 *wbbL*::IS*5*	Lab collection
MAJ286	MAJ1/pDSW204	This study
MAJ330	MAJ1 *frt wbbL+*	[3]
MAJ347	MAJ330 Δ*wecA*::*frt*	This study
MAJ362	MAJ330 Δ*wecB*::*frt*	This study
MAJ484	MAJ330 Δ*gtrB*::*cat*	This study
MAJ557	MAJ330 Δ*wecA*::*frt* Δ*wcaJ*::*frt* Δ*gtrB*::*frt* Δ*arnC*::*kan*	This study
MAJ981	MAJ1 Δ*wecA*::*frt*/pDSW204	This study
MAJ1067	MAJ330 Δ*arnC*::*kan*	This study
MAJ1068	MAJ330 Δ*wcaJ*::*cat*	This study
MAJ1354	MAJ1/pMAJ9	This study
MAJ1385	MAJ557/pDSW204	This study
MAJ1386	MAJ557/pMAJ9	This study
MAJ1677	MAJ1 Δ*wecA*::frt/pMAJ9	This study

Table S3. Plasmids used in this study

Plasmid	Relevant genotype or characteristics	Origin of replication	Source or reference
pB	pBBR1MCS-3 P_{lac} TetR	pBBR1	[4]
pB4[a]	P_{lac}::*S. pneumoniae* serotype 4 capsule locus (*wciI-fnlC*)	pBBR1	[5]
pCP20	λ_{PR}::*flp* λcI857 *bla cat* Rep(Ts)	pSC101	[6]
pDSW204	P_{204} *lacI*^{q} *bla*	pBR	[7]
p*gne*[b]	p*gne* is pMAF12; P_{tac}::*gne* (*C. jejuni*) *lacI*^{q} SpR	IncW	Mario Feldman
pKD3	*bla frt-cat-frt*	R6Kγ	[8]
pKD13	*bla frt-aph-frt*	R6Kγ	[8]
pKD46	P_{araB}::*gam-bet-exo bla* Rep(Ts)	pSC101	[8]
MAJ9[c]	P_{204}::*uppS*	pBR	[9]

[a]Derivative of pB
[b]Derivative of pEXT21
[c]Derivative of pDSW204

Table S4. Primers used in this study

Primer	Sequence[a]	Purpose
P23	ATACTTCTGCTAATAATTTTCTCTGAGAGCATGCATTGTGT GTAGGCTGGAGCTGCTTCG	Δ*wecA*
P24	TGTGTCATCACATCCTCATTTATTTGGTTAAATTGGGGCT ATTCCGGGGATCCGTCGACC	Δ*wecA*
P72	GGATCTTCCCTTACCCCACTGCGGGTAAGGGGCTAATAA CATTCCGGGGATCCGTCGACC	Δ*wcaJ*
P73	CGACAATCGACATCCCCCCGGTCAGCACATTGATAAACT GTGTAGGCTGGAGCTGCTTCG	Δ*wcaJ*
P289	TTGTCTTTAGGGATGCGAAATGAAGATATCTCTTGTAGTT TGTAGGCTGGAGCTGCTTCG	Δ*gtrB*
P290	AAACAAAAGATATATACAATGATACTTTTATTGCTTTATTC ATATGAATATCCTCCTTAG	Δ*gtrB*
P316	GCAAAAACGACGGTTTTCATTATTCATTTTCCTTGCTGGA TGTAGGCTGGAGCTGCTTCG	Δ*arnC*
P317	CATCACAGCCCTTCAGCAACTCGCAGGACAATAAGCCAT GATTCCGGGGATCCGTCGACC	Δ*arnC*
P558	CCCCACTGCGGGTAAGGGGCTAATAACAGGAACAACGAT GTGTAGGCTGGAGCTGCTTCG	Δ*wcaJ*
P559	CCGACCACTTCGCGCCGCTGATGGTTTTTTCACGTAAGC TCATATGAATATCCTCCTTAG	Δ*wcaJ*

[a]All primer sequences are written 5'→ 3'

References

1. Linton D, Dorrell N, Hitchen PG, Amber S, Karlyshev AV, Morris HR, Dell A, Valvano MA, Aebi M, Wren BW: **Functional analysis of the *Campylobacter jejuni N*-linked protein glycosylation pathway.** *Mol Microbiol* 2005, **55:**1695-1703.
2. Kay EJ, Mauri M, Willcocks SJ, Scott TA, Cuccui J, Wren BW: **Engineering a suite of *E. coli* strains for enhanced expression of bacterial polysaccharides and glycoconjugate vaccines.** *Microb Cell Fact* 2022, **21:**66.
3. Rendueles O, Beloin C, Latour-Lambert P, Ghigo JM: **A new biofilm-associated colicin with increased efficiency against biofilm bacteria.** *ISME J* 2014, **8:**1275-1288.
4. Kovach ME, Elzer PH, Hill DS, Robertson GT, Farris MA, Roop RM, 2nd, Peterson KM: **Four new derivatives of the broad-host-range cloning vector pBBR1MCS, carrying different antibiotic-resistance cassettes.** *Gene* 1995, **166:**175-176.
5. Kay EJ, Yates LE, Terra VS, Cuccui J, Wren BW: **Recombinant expression of *Streptococcus pneumoniae* capsular polysaccharides in *Escherichia coli*.** *Open Biol* 2016, **6:**150243.
6. Cherepanov PP, Wackernagel W: **Gene disruption in *Escherichia coli*: TcR and KmR cassettes with the option of Flp-catalyzed excision of the antibiotic-resistance determinant.** *Gene* 1995, **158:**9-14.
7. Weiss DS, Chen JC, Ghigo JM, Boyd D, Beckwith J: **Localization of FtsI (PBP3) to the septal ring requires its membrane anchor, the Z ring, FtsA, FtsQ, and FtsL.** *J Bacteriol* 1999, **181:**508-520.
8. Datsenko KA, Wanner BL: **One-step inactivation of chromosomal genes in *Escherichia coli* K-12 using PCR products.** *Proc Natl Acad Sci USA* 2000, **97:**6640-6645.
9. Jorgenson MA, Kannan S, Laubacher ME, Young KD: **Dead-end intermediates in the enterobacterial common antigen pathway induce morphological defects in *Escherichia coli* by competing for undecaprenyl phosphate.** *Mol Microbiol* 2016, **100:**1-14.
10. Bernatchez S, Szymanski CM, Ishiyama N, Li J, Jarrell HC, Lau PC, Berghuis AM, Young NM, Wakarchuk WW: **A single bifunctional UDP-GlcNAc/Glc 4-epimerase supports the synthesis of three cell surface glycoconjugates in *Campylobacter jejuni*.** *J Biol Chem* 2005, **280:**4792-4802.

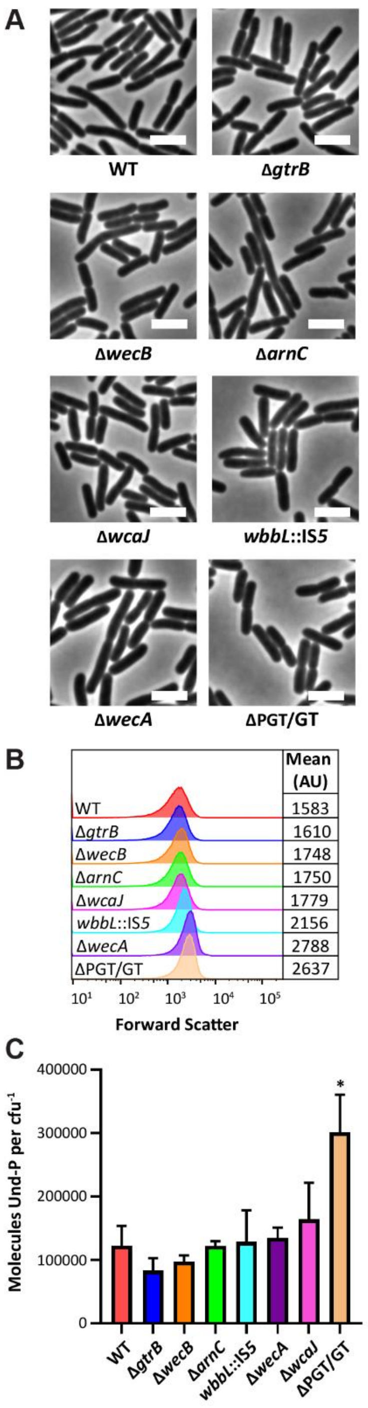

Figure S1. Morphology and Und-P levels in Und-P pathway mutants. (A) Micrographs of cells with the indicated genotypes. Cells were grown in TB at 37°C for approximately 10 doublings until the culture reached an OD600 ~0.4-0.6. The cells were

then imaged by phase contrast microscopy. Bar, 3 µm. (B) Flow cytometry data from live cells in panel A. Histograms of the forward scatter area from 100,000 cells are shown. The mean cell size is shown in arbitrary units (AU). (C) Und-P levels from cells grown in panel A after 3.5 hours. Und-P levels were normalized by dividing Und-P measurements by the mean CFU/ml. Absolute Und-P values are detailed in Table S1. Error bars show +/- standard error of the means. Significance was determined by using an unpaired *t*-test followed by Welch's correction. *$p < 0.05$. Morphological data are representative of two independent experiments. Growth and Und-P measurements are representative of two independent experiments performed in triplicate. The *E*. *coli* strains shown are MAJ330 (WT), MAJ484 (Δ*gtrB*), MAJ362 (Δ*wecB*), MAJ1067 (Δ*arnC*), MAJ1 (*wbbL*::IS*5*), MAJ347 (Δ*wecA*), MAJ1068 (Δ*wcaJ*), and MAJ557 (ΔPGT/GT).

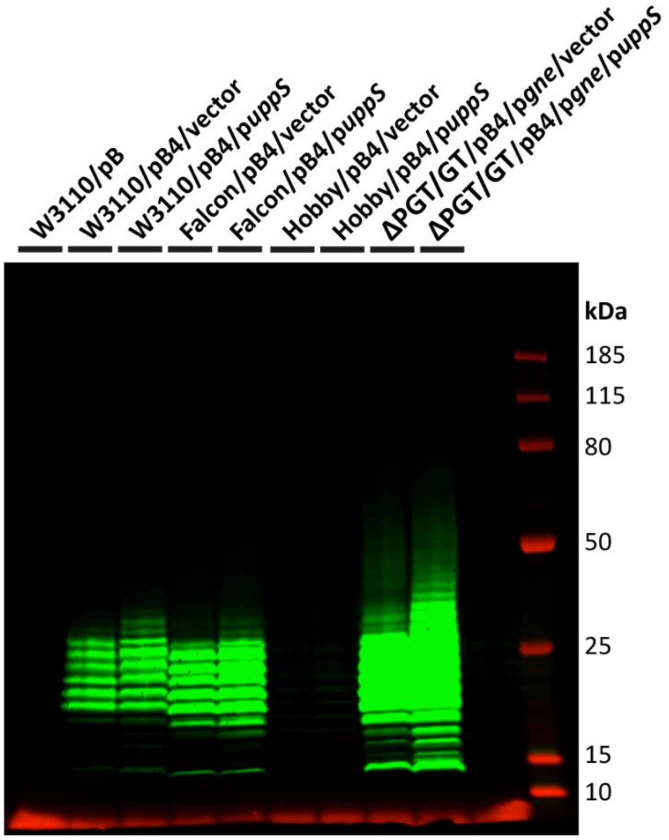

Figure S2. Increasing Und-P levels has little effect on SP4 expression in Falcon and Hobby strains. Western blot showing that increasing Und-P levels (by overexpressing *uppS*) does not have much effect on SP4 expression in Falcon and Hobby cells, which express the *Campylobacter jejuni gne* epimerase from the chromosome [2, 10]. Cells with the indicated genotypes were grown at 28°C in 2YP media for 28 hours. Lysed, whole cell samples were then separated by SDS-PAGE on a 4-12% bis-tris gel and detected using anti-serotype CPS primary and anti-rabbit fluorescent secondary antibody. Results are representative of two independent experiments. The *E*. *coli* strains shown are EJK20 (W3110/pB), EJK2 (W3110/pB4/vector), EJK3 (W3110/pB4/p*uppS*), EJK16 (Falcon/pB4/vector), EJK17

(Falcon/pB4/p*uppS*), EJK18 (Hobby/pB4/vector), EJK19 (Hobby/pB4/p*uppS*), EJK11 (ΔPGT/GT/pB4/p*gne*/vector), and EJK12 (ΔPGT/GT/pB4/p*gne*/p*uppS*).
